# Atypical processing pattern of gaze cues in dynamic situations in autism spectrum disorders

**DOI:** 10.1038/s41598-022-08080-9

**Published:** 2022-03-08

**Authors:** Jia Liu, Jinsheng Hu, Qi Li, Xiaoning Zhao, Ying Liu, Shuqing Liu

**Affiliations:** 1 College of Psychology, Liaoning Normal University, Dalian, 116029 China; 2 College of Basic Medical Sciences, Dalian Medical University, Dalian, 116044 China

**Keywords:** Psychology, Sensorimotor processing, Visual system, Neurological disorders

## Abstract

Psychological studies have generally shown that individuals with Autism Spectrum Disorder (ASD) have particularity in the processing of social information by using static or abstract images. Yet, a recent study showed that there was no difference in their use of social or non-social cues in dynamic interactive situations. To establish the cause of the inconsistent results, we added gaze cues in different directions to the chase detection paradigm to explore whether they would affect the performance of participants with ASD. Meanwhile, eye-tracking methodology was used to investigate whether the processing patterns of gaze cues were different between individuals with ASD and TD. In this study, unlike typical controls, participants with ASD showed no detection advantage when the direction of gaze was consistent with the direction of movement (oriented condition). The results suggested that individuals with ASD may utilize an atypical processing pattern, which makes it difficult for them to use social information contained in oriented gaze cues in dynamic interactive situations.

## Introduction

Autism Spectrum Disorder (ASD) is a complex neurodevelopmental disorder with repetitive behaviors and restricted interests as the core features of social interaction and communication disorders^1^. These symptoms manifest in many areas of people’s development, such as verbal communication, behavioral responses, and establishment of social relationships. Recent studies have shown that individuals with ASD have difficulties in the processing of information containing social meaning, and their cognitive competences are different from others^2^. For many typically developing (TD) individuals, detection of other people's gaze direction, head orientation and other information in social interactions occurs spontaneously^3–6^. These behavioral patterns can help people infer the attentional focus, mental state and behavioral intention of others. However, it is hard for individuals with ASD to process social information in this way. This phenomenon appears early in the development of the disorder as revealed by retrospective studies^7,8^, and it is thought that it may lead to atypical development of social cognition, such as difficulty in comprehending and explaining another’s behavior, as well as guidance of one's own behavior based on such information.

A sizeable literature has been devoted to studying the effect of gaze cues on cognitive processing in individuals with ASD^3,9,10^. Researchers have used social (eye, face orientation) and non-social orientation cues (chair orientation) to explore the orientation adaptation of individuals with ASD and found that they have weaker ability to adapt to orientation cues in social situations^11^. The evidence from eye movement research suggested that individuals with ASD are responsive to gaze as a perceptual cue but ignore its representational meaning. Congiu et al.^8^ used eye-tracking methodology to compare spontaneous gaze following in young children with ASD to that of TD children. The results showed that the processing of gaze cues in children with ASD was mainly driven by perceptual features, such as the position of the irides in the sclera, rather than information about social significance provided by gaze cues. These results echoed the deficit in understanding the social meaning of social cues that was identified by Klin, Jones, Schultz, Volkmar, & Cohen^12,13^. Klin et al.^13^ analyzed the visual scanning paths of the adult viewers with autism to verbal and non-verbal cues in a social scene from a film. The results suggested that the viewer with ASD responded primarily to the verbal cue and neglected the non-verbal gesture. They also found that whereas TD viewers converge on the eye region, some individuals with ASD converge on the mouth regions, and other individuals with ASD converge on peripheral to the face^13^. These studies suggested that individuals with ASD have an atypical processing pattern for gaze cues.

In real life, gaze cues are often associated with other social movements such as approach and escape, but previous studies have rarely put these cues into some type of dynamic situation. Instead, gaze cues are presented as static or abstract images as well as simple displays of head spinning^14,15^. Therefore, we used a special experimental paradigm called chase detection, in which gaze cues are added to moving subjects. In this paradigm, originally designed by Gao, Newman and School^16^, a ball (referred to as ‘wolf’) is chasing another (referred to as ‘sheep’) and this chasing behavior was defined by the degree to which the wolf reliably moved in the direction of the sheep. Accuracy for identifying chasing behavior can be evaluated under different levels of difficulty by manipulating chasing subtlety (set by adjusting the angular deviation of the heading of the wolf relative to the sheep) and three different orientation gaze cues to explore their influence on chase detection by TD individuals. The results showed that accuracy was significantly higher under oriented gaze cue conditions than under perpendicular and reverse cue conditions. The performance advantage generated by the alignment of gaze direction and movement direction is called the oriented advantage, and it is considered one of the typical processing patterns of gaze cues in dynamic situations.

By adding gaze cues to the paradigm, Vanmarcke and his colleagues recently tested the role of social interpretations of adolescents with and without ASD^17^. Their results showed that the ASD group was less accurate than the control group and the accuracy of both groups was better in the social condition than in the non-social condition, but there was no interaction between group and condition. This may indicate that adolescents with and without ASD did not differ in their use of the added social or non-social cues. The intact performance of adolescents with ASD in this study seems inconsistent with the view that individuals with ASD have an atypical processing pattern for gaze cues.

The theory of enactive mind (EM) offers an approach to social cognitive development intended to explain how individuals with ASD search for meaning when presented with naturalistic social scenes^18^. It proposes a developmental hypothesis of autism in which the process of acquisition of embodied social cognition is derailed early on as a result of reduced salience of social stimuli and concomitant enactment of socially irrelevant aspects of the environment. Further, individuals with ASD seem to be fully capable of collecting social information, but cannot learn the social meaning represented by the information nor how to translate it into socially adaptive behaviors^12,13^. Based on this, we speculate that the reason why social cues had a positive effect on the performance of the participants with ASD in the Vanmarcke et al. study may be due to their distinctive processing mode of social cues. In other words, these cues may have physical, but not social, implications for individuals with ASD. In addition, the design of Vanmarcke et al. experiment was unable to explore the processing patterns of individuals by comparing their performance to gaze cues in different directions. It is a computer-based behavioral experiment, unable to further explore other indices related to potential processing mechanisms.

Therefore, we wanted to explore the processing pattern of gaze cues in individuals with ASD by using chase detection paradigm and eye-tracking methodologies. We mainly focused on two questions in our experiment. The first was whether there is a difference between the processing mode of gaze cues in individuals with or without ASD in a dynamic chasing context. The second question examined the influence of a variety of gaze cues on visual processing and cognitive processing in the chase detection process. We used three kinds of gaze cues: oriented, reverse, and perpendicular.

This not only ensured that the physical characteristics are highly similar, but also enabled us to identify differences in the processing patterns of the participants through the comparison of the cues. To be specific, all three conditions are advantageous to chasing detection and have directivity if the participants did not regard them as social information. For example, let’s assume that the participants imagined a motion ball with the reverse cues, which can be seen as something like a rocket, and the two red dots at the back of the ball are like the tail fin of the rocket. If the participants had a typical processing mode for the gaze cues, we predicted that participants: (1) could understand the social information contained in gaze cues, (2) would pay more attention to where the gaze cues are pointing, and (3) oriented gaze cues would be effective cues for participants to detect the chase, while reverse and perpendicular gaze cues would hinder detection. However, if participants had an atypical processing mode, they would not have the oriented advantage. In addition, to further explore the participants' visual and cognitive processing, eye-tracking methodology was combined with the behavioral assay. This study assumed that participants with ASD use an atypical processing pattern in the chase detection task, consistent with the viewpoint of EM.

## Method

### Participants

Twenty adults with ASD were recruited for this study (4 females and 16 males). We excluded two males with ASD because they failed to pass the calibration procedure. Thus, the final sample consisted of eighteen adults with ASD. They were recruited from the Aina autism service center in Dalian. All diagnoses were completed at qualified hospitals (e.g., Peking University Sixth Hospital). Six participants with ASD had their diagnosis confirmed by the Autism Diagnostic Interview-Revised (ADI-R) and twelve of them were confirmed by the Autism Diagnostic Observation Schedule (ADOS-2). The control group consisted of eighteen individuals recruited through bill-posting (4 females and 14 males, mean age = 19.51, *SD* = 0.98). IQ was measured using the Chinese version of Wechsler Adult Intelligence Scale—Third Edition (WAIS-III;^19^). Detailed descriptions of participant characteristics can be found in Table 1. There were no significantly differences between the ASD and control groups in chronological age, verbal IQ, performance IQ, and full-scale IQ. All participants had normal or corrected-to-normal vision.

**Table 1:** Participant characteristics for ASD and TD groups.

		ASD group	TD group	*Statistic*	*p*
*N*		18	18		
Age(years)	Mean	20.84	19.51	*t* (34) = 1.62	0.11
	SD	3.36	0.98		
Gender ratio	Females	4	4	^χ2 ^(1) = 0.00	1.00
	Males	14	14		
WAIS IV Verbal IQ	Mean	96.01	102.22	*t* (34) = 1.86	0.07
	SD	11.25	8.61		
WAIS IV Performance IQ	Mean	106.78	109.28	*t* (34) = 0.99	0.33
	SD	8.43	6.70		
WAIS IV Full Scale IQ	Mean	105.01	108.22	*t* (34) = 1.12	0.27
	SD	9.33	7.83		

The research was conducted according to the principles of the Declaration of Helsinki and was approved by the Ethical Committee of School of Psychology at Liaoning Normal University. We checked whether the participants with ASD were capable of understanding the task requirement through interviews. We obtained written informed consent from all participants and their parent and/or legal guardian. They were debriefed and thanked following their participation.

### Material

**Figure 1:**
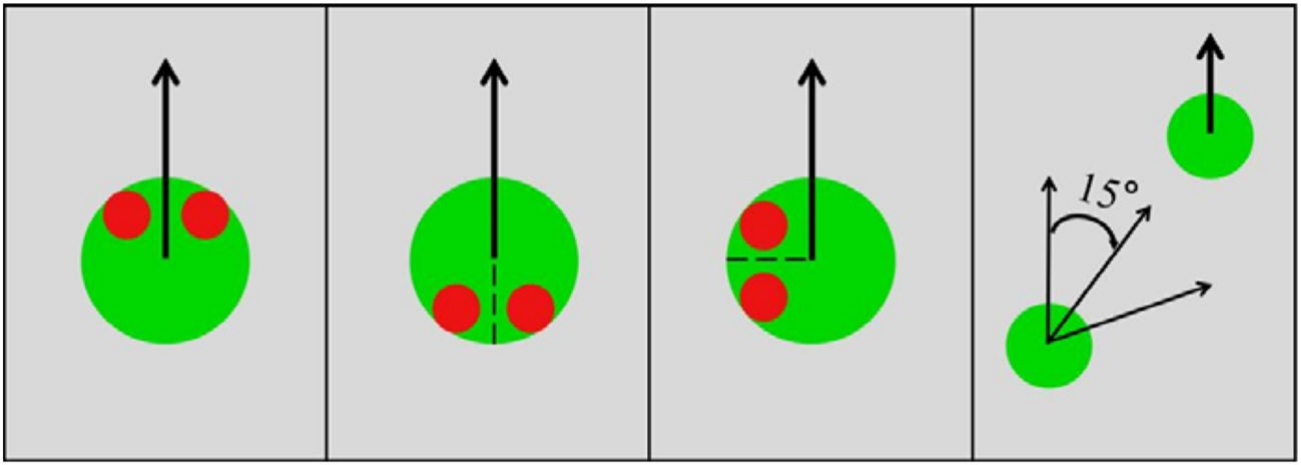
Illustration of the three cue conditions and chasing subtlety. The left image shows oriented cues, the center left one shows reverse cues, the center right image shows perpendicular cues, and the right image shows 15° chasing subtlety. The black arrows represent the direction of movement of the balls.

The displays were made by Matlab2014a and presented on a PC computer. Each of them contained four green balls (apparent size: 1° of visual angle) with two red eyes shown for 10,000 ms. We made references to the experimental material design of Gao et al.^16^ and Vanmarcke et al.^17^. At the start of each trial, three of the balls started moving at a constant speed of 14.5°/s and moved haphazardly by randomly changing direction within a 120° window (approximately every 170 ms). The fourth ball was termed the ‘wolf ’ and moved differently. The heading of the wolf towards the sheep (the ball being chased), was manipulated by altering its maximal angular deviation (chasing subtlety). We set three different chasing subtleties: 15°, 45°, and 75°. For example, when the chasing subtlety was 15°, the wolf could move in any direction within a 30° window which always centered on the sheep (Fig. 1). To maintain a similar movement pattern, the wolf chased an invisible ball in chase-absent trials, whereas it chased one of the three visible balls in chase-present trials. Furthermore, the chasing behavior of the wolf was delayed for 170 ms at the start of each trial to make the chasing appear smoother.

We also set three conditions. In oriented conditions, the direction of red eyes always matched the directions in which they were moving. In reverse conditions, the balls moved in opposite direction to the direction of red eyes. In perpendicular conditions, the orientation of the eyes was always perpendicular to the direction of the balls’ motion (Fig. 1).

### Procedure

The displays were presented on a 21­inch DELL computer with a 60 Hz refresh rate and 1024 × 768 pixel resolution, and were viewed from 60 cm in a dimly lit room. Eye movements were recorded monocularly by Eyelink 1000 with a sampling rate of 1000 Hz and a spatial resolution of 1°. Each participant was instructed to position their chin in a chin rest to hold their head stationary and to avoid blinking as much as possible during each trial.

Before each block, participants completed a five-point calibration phase. After successful completion of the calibration phase, they were told to observe the stimuli displayed on the screen. An experimenter operated the eye tracker from a laptop computer not visible to the participants.

The test phase included 3 blocks (conditions) and took about 30–40 min. All participants completed 39 trials per condition (13 per chasing subtlety, of which 10 were chase-present and 3 were chase-absent trials). Then participants completed a training phase to make sure they comprehended the instructions. After each trial, they were given visual feedback. Training ended when the participant responded correctly in 6 consecutive trials. These trials were not included in the formal analysis.

**Figure 2:**
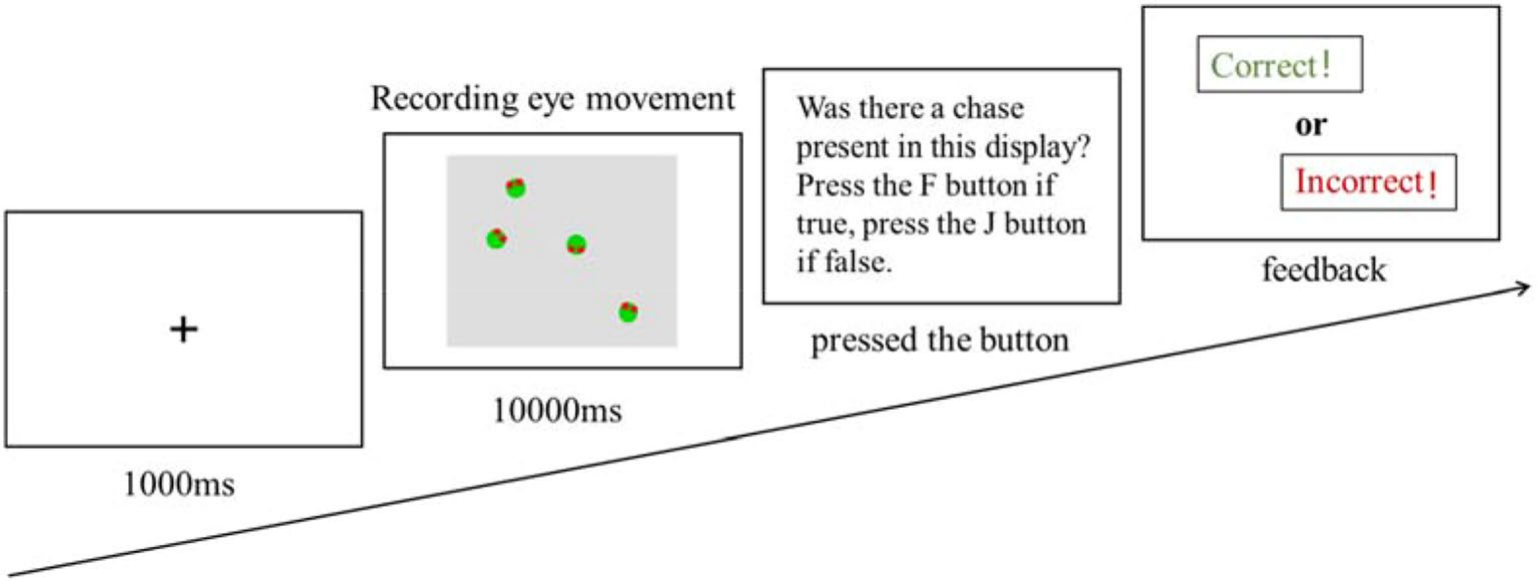
Graphical overview of the trial design.

All instructions were provided on the computer screen, and every trial started with a 1-s fixation display before the chasing display was presented (Fig. 2). The eye tracker recorded the participant’s eye movement during the 10-s animation. At the end of each trial, the participants were required, without time constraints or feedback, to indicate whether or not the trial contained a chase. All participants had to complete all three blocks presented in a counterbalanced order.

### Data analysis

**Figure 3:**
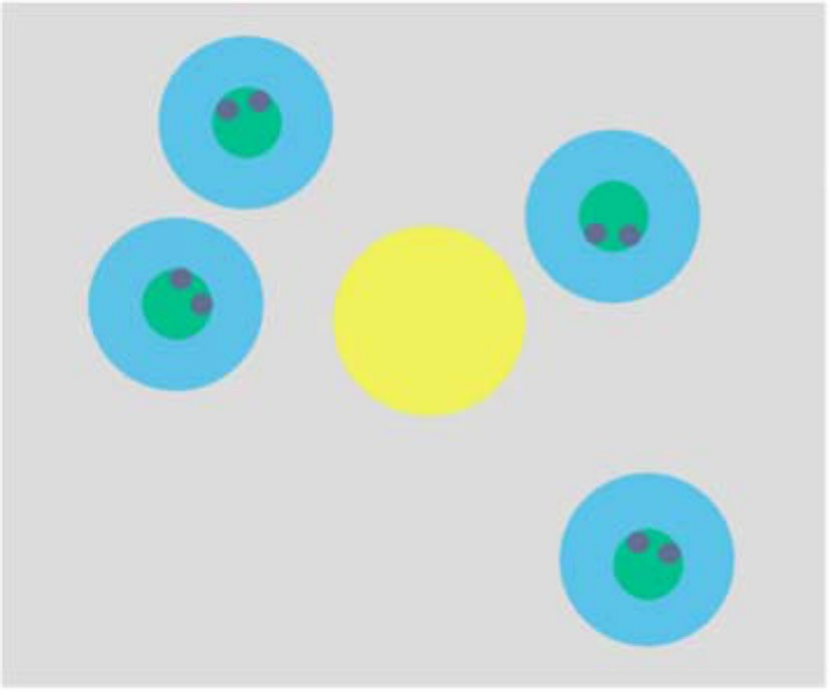
Samples of AOIs. The four blue areas were AOI1, which were located within 2.5 times the radius of every agent. The yellow area was AOI2, which was located within 2.5 times the radius of agent at the barycenter of 5 agents.

Some researchers suggested that two visual exploration strategies which are used by participants to detect a chase: either following 1 agent for a certain amount of time (and jumping to another agent until a chase is detected), or looking roughly at the barycenter of all four agents, thus obtaining an optimal view of the movements of all agents simultaneously^20,21^. To determine the strategies of participants through eye movement data, we identified two dynamic areas of interest (AOIs) on each display for analysis (See Fig. 3). Based on these AOIs, we calculated five indicators: agent-looking rate, barycenter-looking rate, stray-looking rate, ocular sensitivity and cognitive sensitivity.

Agent-looking rate was defined as the ratio of fixation count of AOI1 (FC1) to the sum of FC1 and FC2. Barycenter-looking rate was defined as the ratio of fixation count of AOI2 (FC2) to the sum of FC1 and FC2. Stray-looking rate was defined as the proportion of gaze falling anywhere else (excluding AOI1 and AOI2). Because agent- and barycenter-looking rate are complementary, we analyzed all indicators except barycenter-looking rates.

We also used a previously described measure related to the distribution of gaze across the 4 agents: the agent preference index (API), defined as the standard deviation (SD) of looking rates on each of the 4 agents^22^. The idea is that if participants detect the chase, they will tend to track the sheep and the wolf and, hence, will show unevenly distributed looking rates across agents and a high SD. In contrast, if they do not detect the chase, all agents should have an equal probability of being tracked, and the SD should be lower. Thus, API provided a measure of participants’ implicit detection of chasing, independent from the explicit response. Two additional sensitivities were derived from the API using the same signal detection approach as used for the chasing detection sensitivity. Ocular sensitivity measures the extent to which the API reveals the implicit detection of chasing. Cognitive sensitivity measures the extent to which explicit chase responses reflect the implicit detection of chasing. Cognitive sensitivity is thus more related to high­level decisional processes about intentional information. This was calculated as:

$${\text{Sensitivity}}:{\text{d}}^{^{\prime}} = {\text{ z}}\left( {\text{H}} \right) - {\text{z}}\left( {\text{F}} \right)$$

H is the hit (correctly detecting a chase) rate, F is the false-alarm (saying a chase exists when one did not) rate and z is the inverse function of the normal distribution. Null values of H and F were replaced by 1/2 N, with N being the number of trials per participant (39). When H and F were equal to 1, they were replaced by 1 − 1/2 N.

## Result

### Behavioral data

Total fixation duration of AOI1 and AOI2(s) that were less than 3 SD above their group’s mean were excluded from the analysis (2.98% of trials of ASD group, 1.71% of trials of TD group). Based on the dichotomous nature of the dependent variable, we analyzed the accuracy (correct/incorrect) scores by using multilevel logistic regression (MLR) model^23^. In the model, participants were specified as random intercepts. Chasing subtlety and type of gaze-cues were specified as crossed random effects with random slopes. Group, chasing subtlety and type of gaze-cues were treated as fixed factors. Table 2 illustrated the statistical estimates of the fixed effects and random effects of accuracy scores in MLR model. The results showed that the main effects of the groups and chasing subtlety were significant. Compared with TD group [mean = 0.85 *SD* = 0.08], ASD group had lower accuracy [mean = 0.72 SD = 0.19] (*p* < 0.001). Both groups’ accuracy in 15° condition [mean = 0.87 *SD* = 0.14] was higher than 45° condition [mean = 0.84 *SD* = 0.12] (*p* = 0.017) and 75° condition [mean = 0.65 *SD* = 0.17] (*p* < 0.001).

**Table 2:** Fixed effects and random effects of accuracy scores.

	Fixed effects			Random effects	
	*b*	*z*	*p*	Variance	Std. Dev
(Intercept)	5.42	5.58	< 0.001	4.17	2.04
Condition	0.35	0.89	0.37	0.09	0.3
Subtlety	–1.31	–3.72	< 0.001	0.46	0.68
Group	–2.64	–2.33	0.02		
Condition: subtlety	–0.24	–1.67	0.09	0.01	0.11
Condition: group	–0.68	–1.61	0.11		
Subtlety: group	0.62	1.38	0.14		
Condition: subtlety: group	0.35	2.12	0.03		

**Figure 4:**
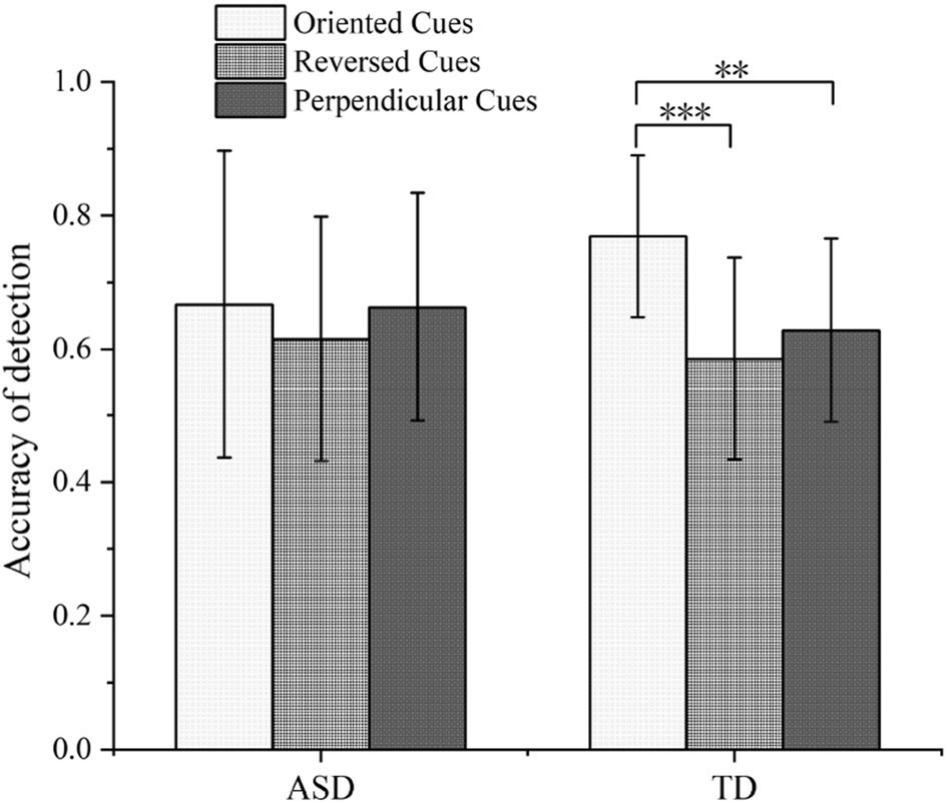
Accuracy of detection of the two groups in the 75° chase subtlety condition. *Note* ** *p* < 0.01, *** *p* < 0.001.

The interaction of all three factors was significant. Post hoc analyses showed that in the 15° and 45° chase subtlety conditions, the accuracy of the TD group was higher with all types of gaze-cues than the ASD group. However, the accuracy of the two groups was similar in the 75° condition. Moreover, in the 75° chase subtlety condition, the TD group’s accuracy with oriented cues [mean = 0.77 *SD* = 0.12] was higher than with reversed [mean = 0.59 *SD* = 0.15] (*p* < 0.001) or perpendicular cues [mean = 0.63 *SD* = 0.14] (*p* = 0.007), whereas the ASD group’s accuracy showed no significant difference between the three types of gaze cues (Fig. 4).

### Eye movement data

Data were analyzed by linear mixed models (LMM) using the lme4 package in R^24^. In the models, participants were specified as random effects with both random intercepts and random slopes. Group, chasing subtlety and type of gaze-cues were treated as fixed factors. Means for the eye movement data are shown in Table 3, and the fixed effects estimates for these data are shown in Table 4. The results showed that the effects of group were significant. Compared with the TD group, the ASD group had higher agent-looking rate, stray-looking rate, ocular sensitivity, and lower cognitive sensitivity (|*t*|s > 5.40, *p*s < 0.001). In other words, the two groups had different eye movement patterns during chasing detection, indicating that their processing methods are different.

**Table 3:** Means with standard deviations in parentheses of eye movement measures for the two groups.

Eye movement data	ASD group	TD group
Agent-looking rate	0.64 (0.04)	0.52 (0.06)
Stray-looking rate	0.48 (0.11)	0.39 (0.04)
Ocular sensitivity	1.25 (0.35)	1.05 (0.28)
Cognitive sensitivity	0.67 (0.29)	0.87 (0.26)

**Table 4:** Fixed effect estimates of eye movement measures for the two groups.

Eye movement data	Group effect (ASD vs. TD)			
	*b*	*t*	*p*	95% CI
Agent-looking rate	0.11	16.89	< 0.001	[0.10, 0.13]
Stray-looking rate	0.09	9.36	< 0.001	[0.07, 0.11]
Ocular sensitivity	0.20	5.41	< 0.001	[0.13, 0.28]
Cognitive sensitivity	− 0.20	− 5.74	< 0.001	[− 0.27, − 0.13]

*CI* Confidence Interval.

**Figure 5:**
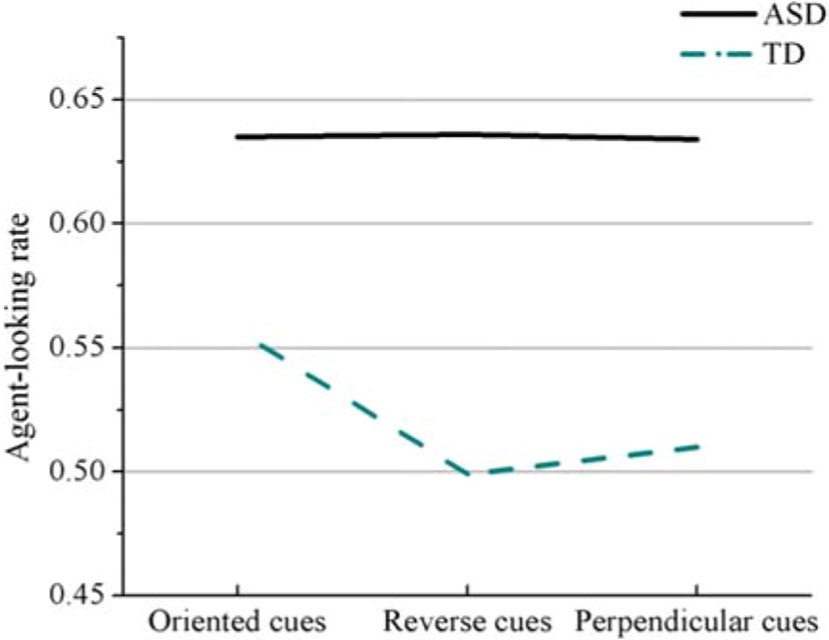
Agent-looking rate of the two groups for the three types of gaze cues.

The interaction between gaze-cues and group factors was significant for agent-looking rate (*b* = 0.02, *t* = 2.90, *p* = 0.004, 95% CI = [0.01, 0.04]), stray-looking rate (*b* =  − 0.07, *t* =  − 2.15, *p* = 0.03, 95% CI = [− 0.13, − 0.01]), and ocular sensitivity (*b* = 0.29, *t* = 2.48, *p* = 0.01, 95% CI = [0.06, 0.53]). But it was not significant for cognitive sensitivity (*t* =  − 0.93, *p* > 0.05). Further analyses showed that the ASD group’s agent-looking rate for the three kinds of gaze-cues were all higher than the TD group. In the TD group, agent-looking rates with oriented cues [mean = 0.56 *SD* = 0.07] were higher than with reversed cues [mean = 0.50 *SD* = 0.08] (*p* < 0.001) or perpendicular cues [mean = 0.51 *SD* = 0.07] (*p* < 0.001). However, there was no significant effect of gaze-cue in the ASD group (Fig. 5).

**Figure 6:**
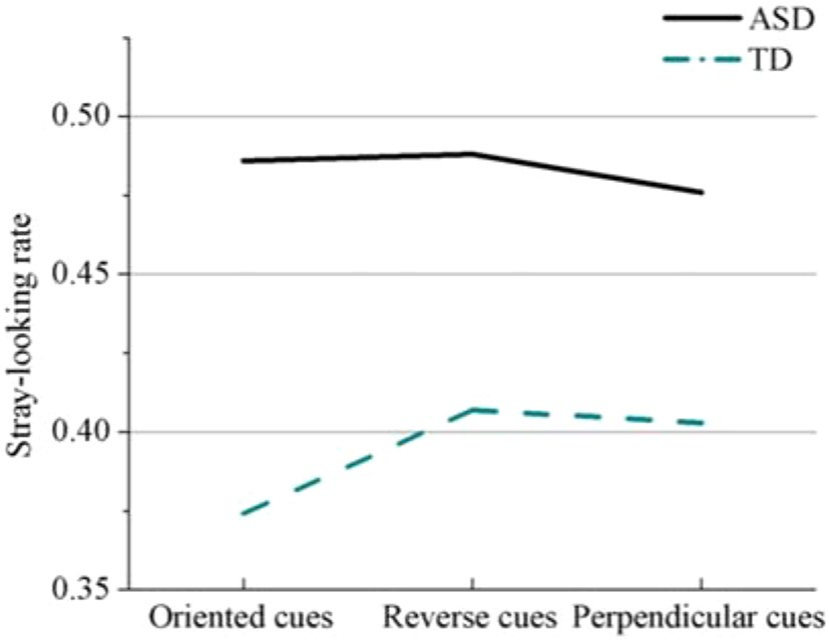
Stray-looking rate of the two groups for the three types of gaze cues.

The ASD group’s stray-looking rates for the three kinds of gaze cues were significantly higher than the TD group. For the TD group, stray-looking rates with oriented cues [mean = 0.37 *SD* = 0.04] were lower than with reversed cues [mean = 0.41 *SD* = 0.04] (*p* = 0.006) or perpendicular cues [mean = 0.40 *SD* = 0.05] (*p* = 0.004). However, there were no significant cue-related effects on the stray-looking rates of the ASD group (Fig. 6). The ASD group’s ocular sensitivity to oriented cues [mean = 1.31 *SD* = 0.39] was significantly higher than that of the TD group [mean = 1.09 *SD* = 0.28] (*p* = 0.004), and their ocular sensitivity to perpendicular cues [mean = 1.32 *SD* = 0.38] was significantly higher than that of the TD group [mean = 1.00 *SD* = 0.29] (*p* < 0.001). But there were no group differences for the reversed cues.

**Figure 7:**
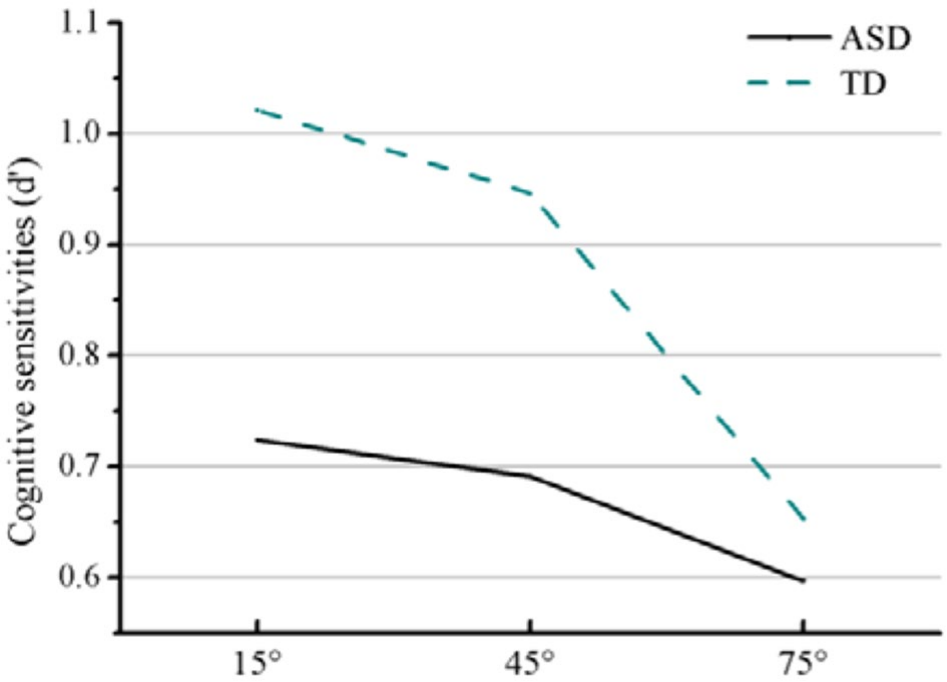
Cognitive sensitivity of the two groups in the three chase subtlety conditions.

The interactions of group × chasing subtlety was significant for cognitive sensitivity (*b* = 0.12, *t* = 2.99, *p* = 0.003, 95% CI = [0.04, 0.20]). Further analyses showed that the ASD group’s cognitive sensitivity [mean = 0.72 *SD* = 0.32] was lower than the TD group [mean = 1.02 *SD* = 0.28] (*p* < 0.001) in the 15° condition. In the 45° condition, the ASD group’s cognitive sensitivity [mean = 0.69 *SD* = 0.30] was lower than the TD group [mean = 0.95 *SD* = 0.28] (*p* < 0.001), but not in the 75° condition (Fig. 7).

## Discussion

In this study, the chase detection paradigm was used to compare the way that individuals with and without ASD processed gaze cues in dynamic interactive situations, and the eye-tracking method was used to explore the impact of gaze cues on their visual and cognitive processing during detection.

Our behavioral data show that in the low (15°) and moderate (45°) chasing subtleties, the influence of directional gaze cues did not appear to have obvious effects on accuracy of detection of either group. Under the high (75°) chasing subtlety, on the other hand, participants were influenced more by gaze cues. This suggests that when the task is of moderate difficulty, individuals will rely more on agents themselves to detect the chase, such as the way they move and how far away they are. When the chase subtlety cues provide less helpful information to accurately detect a chase, the role of gaze cues will be revealed (which was the purpose of setting the chasing subtleties). Chasing subtlety is the most significant factor affecting the accuracy of chase detection. With the increase of chasing subtlety, the accuracy of both groups decreased. Therefore, the effect size of the main effects of group is smaller than the main effects of chasing subtlety.

Further analysis shows that when the chasing subtlety is high, the accuracy of the TD group under the oriented condition is significantly higher than that under the reverse or perpendicular cue conditions. These participants showed a detection advantage under oriented conditions, which was similar to previous studies^25,26^. In contrast, the ASD group showed no detection advantage under the oriented condition. When the chasing subtlety was high, there was no significant difference in their accuracy between the oriented condition and the other two gaze cue conditions, which is consistent with our hypothesis.

Defects of executive functions, such as response inhibition and poor cognitive flexibility, will affect keyboard responses of the ASD group, thereby preventing behavioral data from revealing their processing pattern. To overcome this bias, we collected eye movement data during chase detection trials. These data were incorporated into the working model of chase detection described by Roux and his colleagues^22^ to further comprehend detection processing of ASD participants.

According to this model, the process of chase detection includes two aspects. The first is attention to the stimulus, which is mainly represented by the stray-looking rate describing the effects of low-level eye movement factors. The second is exploration, which includes the visual response stage and the behavioral response stage. Visual responding is measured by ocular sensitivity, which represents the extent to which the API reveals the implicit detection of chasing. The behavioral response stage is measured by cognitive sensitivity which shows the extent to which explicit chase responses reflect the implicit detection of chasing. In addition, an individual’s exploration strategies are mainly determined by their agent-looking rates. Accordingly, we will discuss the influence of gaze cues on the two stages of chase detection processing.

Previous studies have provided evidence of how eye movement patterns reflect global processing strategies^27–29^. In our eye-tracking data analysis, the proportions of each participant’s eye gaze located within different areas of interest were calculated (agent-looking rate and stray-looking rates). We noticed that when TD participants detected the chase among four moving balls, they focused more on the central area of the balls rather than on individual balls. This suggested a processing strategy whereby TD participants tended to integrate multiple stimuli by paying close attention to their displacement relationships with each other. Under the oriented condition, the agent-looking rates of the TD group were significantly higher than in other conditions. This suggested that the oriented gaze cues helped the TD group detect chasing by enabling the participants to direct their eye gazes more to each agent than on the center of the agents.

For the ASD group, the agent-looking rate was approximately 0.63, significantly higher than that of the TD group, whether under the oriented, perpendicular or reverse cue conditions. This suggests that young adults with ASD are less likely to adopt the integral detecting strategy and more inclined to locate and process agents separately, regardless of the nature of the cue. These participants were insensitive to the gaze cues and were not good at adjusting detection strategies according to the different directions of the gaze cues. Our finding is consistent with a weak central cognitive processing style, which refers to the detail-focused processing style proposed to characterize ASD^30,31^.

This viewpoint was also supported by the behavioral results showing that the chase detection accuracy of the two groups decreased as the difficulty of the chase subtlety increased, and the TD group had a greater reduction than the ASD group. This difference indicates that chase detection performance of the ASD group was less dependent on chasing subtlety than that of TD. These results were similar to those of Vanmarcke et al.^17^. They suggested that the processing of the motion relationship between agents has lost its saliency with higher chasing subtlety and a more attention-focused processing of each of the moving balls gradually became a better search strategy.

Previous studies in the field of social attention have shown that individuals with ASD process social information differently from TD individuals. Vlamings et al.^32^ examined reflexive visual orienting following eye direction or symbolic (arrow) cues, either congruent or incongruent with target presentation, in persons with and without ASD. These investigators found that both the incongruent eye direction cues and the arrow cues enabled individuals with ASD to detect the direction of a target more quickly. This indicates that, unlike TD individuals, individuals with ASD might not possess a specialized module for the processing of gaze cues. Other researchers found that eye gaze cues attracted attention more effectively than the arrow in TD children, while children with ASD shifted their attention equally in response to eye gaze and arrow direction, suggesting they failed to show preferential sensitivity to the social cue^33^. Combining behavioral and eye movement data, we guessed that the TD group socialized the gaze cues by focusing more on where the gaze is pointing. In the oriented condition, the ball was chased in the range indicated by the line of sight, which helped TD participants detect a chase. However, under the reverse and perpendicular conditions, the range of the line of sight was not followed by the chased ball, and the inconsistency between the line of sight and the direction of motion violated their social perception^11,34,35^. Therefore, their detection accuracy was lower under these two conditions. But individuals with ASD are different from them, and there may be no differences in processing patterns of social cues and non-social cues.

Moreover, Šimkovic and Trauble^36^ explored the role of eye movements in the detection of chasing. They argued that subjects do not compare the movement of the pursued pair to a singular template that describes a chasing motion. Rather, subjects bring certain hypotheses about what features of motion may qualify as chase and then, through feedback, they learn to look for a motion pattern that maximizes their performance. However, we did not observe this gradually optimized processing strategy in the eye movement data of the ASD group whose agent-looking rates were comparable in all gaze cue conditions. This indicated that their visual detection strategy was relatively stable and unaffected by gaze cues. This result also confirmed the EM hypothesis that individuals with ASD have difficulty changing their strategies to adapt to a change in the environment^18,37^.

The results of sensitivity analysis showed that the ocular sensitivity of participants with ASD was generally higher than that of the TD group, while the cognitive sensitivity was generally lower. It revealed that the implicit, early and online detection of chasing was intact in participants with ASD. Their eye movements were more related to the presence of a chase, suggesting that they may have more often correctly processed chase information and detected chasing at the ocular level. Furthermore, their preferred local looking strategy partly explained the increased ocular sensitivity. The decreased cognitive sensitivity revealed difficulties deciding whether a chase was present or not and/or producing the appropriate response, even when their eye movement patterns reveal that the chasing information had been correctly processed at the visual level^22^. The research of Sevgi et al.^38^ supports our result. They considered that autistic traits may not impair the ability to process social information per se, but rather by a low weighting or precision of social cues during decision-making.

Although this study supports the hypothesis that the processing of gaze cues in individuals with ASD is atypical, there remain limitations in this study that should be addressed in future research. One limitation of this study is the small sample size. According to the effect size of this experiment, we made a post hoc power analysis by using G-Power 3.1.9.7. The result showed that 92.74% power was achieved with the final sample size of 36 participants for the experiment. Nonetheless, it is necessary to replicate the results in a larger sample. In this experiment, there were only four females in the ASD group, making it difficult to conduct a meaningful statistical analysis of gender differences. The intensity of ASD symptoms and comorbidities should be further evaluate with other instruments.

In addition, Genearalised Additive Multilevel Models (GAMMs) would be a much more precise and informative way to analyse gaze trajectories within trials. GAMMs allow for analysis of continuous variables and nonlinear interactions. This is an advantage for analysis of fixation data, as processing is often influenced by continuous predictors, such as time^39^.

Studies have shown that in TD individuals, the ability to estimate whether an object is social by motor cues appears early in infancy, but this ability is deficient in individuals with ASD^2,12,40^. The different performance of their social cognition may result from a severe lack of social informational input at an early stage, leading to insufficient social informational processing needed to promote the development of corresponding systems within the nervous system^41,42^. Participants selected for this study were younger adults with ASD because the chase detection paradigm requires higher attention and comprehension of participants. Children with ASD may be less likely to understand task requirements or unable to maintain a longer period of concentration^43,44^. Therefore, future studies should be designed with more accessible and understandable experimental tasks to explore the causes of social cognitive abnormalities in children and adolescents with ASD, if possible, utilizing a developmental perspective.

In addition, in this study, eye-tracker technology was used to record the subject's eye movements during the behavioral experiment. This method enabled us to distinguish their processing strategies at different phases of the task. Our findings show the value of eye movement technology in exploring dynamic interactive social processing-related problems of individuals with ASD. Using neurophysiological recording methods such as ERP and fMRI to further understand the neural mechanism and physiological activation of individuals with ASD may also be good ways to explore their processing of social information.

In conclusion, this study supports the hypothesis that the role of a variety of gaze cues in the chase detection process of individuals with ASD was significantly different from that of TD individuals. In contrast to TD participants, individuals with ASD utilize an atypical processing pattern, which makes it difficult for them to use social information contained in oriented gaze cues. In addition, there was a large discrepancy between their visual and behavioral responses. These findings may provide new evidence for the EM theory that individuals with ASD can process social information, but they cannot learn the social meaning represented by the information nor how to translate it into socially adaptive behaviors.
